# Cyberbullying definitions and measurements in children and adolescents: Summarizing 20 years of global efforts

**DOI:** 10.3389/fpubh.2022.1000504

**Published:** 2022-10-25

**Authors:** Wei Zhang, Shiqing Huang, Lawrence Lam, Richard Evans, Chengyan Zhu

**Affiliations:** ^1^School of Medicine and Health Management, Tongji Medical College, Huazhong University of Science and Technology, Wuhan, China; ^2^School of Information Resource Management, Renmin University of China, Beijing, China; ^3^Faculty of Medicine, Macau University of Science and Technology, Macau, China; ^4^Faculty of Medicine and Health, The University of Sydney, Sydney, NSW, Australia; ^5^Faculty of Health, University of Technology Sydney, Sydney, NSW, Australia; ^6^Faculty of Computer Science, Dalhousie University, Halifax, NS, Canada; ^7^School of Political Science and Public Administration, Wuhan University, Wuhan, China

**Keywords:** cyberbullying definition, cyberbullying measurement, systematic review, children and adolescents, ASReview

## Abstract

Despite numerous instruments existing to assist in the measurement of specific cyberbullying behaviors or cyberbullying in general, it is still unclear their purpose, corresponding scenarios, and their effectiveness. This study, therefore, aims to provide a comprehensive review of academic efforts on cyberbullying definitions, measurements, and their effectiveness in children and adolescents in the past two decades. A systematic review was performed using ASReview, an open source machine learning systematic review system. Three bibliographic citation databases, including Web of Science core collection, PubMed, and EBSCO were adopted for all relevant literature published from January 2001 to August 2021. In total, twenty-five studies, mentioning seventeen cyberbullying measurement scales, met the study collection criteria. The results found that most failed to provide a clear definition of cyberbullying, often providing unclear and inconsistent descriptions for the youth. Similarly, studies found it difficult to clearly reflect the three key elements of bullying, namely: harmfulness, repetitiveness, and the power imbalance between bullies and victims. With regard to cyberbullying types, most presented two or three categories, including victimization, perpetration, and bystanding, while some suggested four types based on the nature of the cyberbullying behavior, including written or verbal, visual or sexual, character impersonation, and exclusion. If characteristics are considered, cyberbullying becomes more specific with multiple categories being proposed, including flaming (or roasting), harassment, denigration, defamation, outing, jokes, online sexual harassment, and cyberstalking. With regard to measurements, many scales have been proposed and frequently refined to capture specific cyberbullying experience of the youth. This study emphasizes the value and importance of providing clear cyberbullying definitions and helps scholars in youth cyberbullying choose appropriate measurement scales.

## Introduction

Advancements in social media and other internet-enabled technologies have led to a constantly connected world where global citizens converse, share experiences and build relationships. Despite such advantages, a dark side to the internet has gradually evolved. Unlike traditional bullying, such as verbal and physical abuse, which has experienced a slow decline, globally, cyberbullying is quickly growing. Olweus (1) suggested three key elements of cyberbullying: harmfulness, repetitiveness, and the power imbalance between bullies and victims. Accordingly, definitions of cyberbullying are expected to reflect these three elements using the cyber medium (2). However, its definition has garnered considerable scholarly attention in recent years with significant debate on how it is defined. In this study, we define cyberbullying as behaviors expressed by an individual or group through Information and Communication Technologies (ICT), such as social media and e-mail, that repeatedly convey hostile or offensive information with the intention of causing harm or discomfort to others (3, 4).

In the last two decades, the emergence of social digital technologies has dramatically changed how society communicates and has significantly shaped the act of cyberbullying. Prior research on cyberbullying has highlighted numerous methods that bullies use to attack victims in cyberspace, such as flaming (or roasting), harassment, character imitation, deception, exclusion, slander, and cyberstalking. Due to the growing popularity of social media platforms, such as Facebook, there has been a recent resurgence in academic and practitioner research on cyberbullying (5, 6). A study published in 2015, when social media was growing exponentially, found that the prevalence rate of cyberbullying was around 23%. In 2021, an updated systematic review reported that the prevalence of cyberbullying among children and adolescents was on the rise, suggesting a cyberbullying perpetration rate of 25.03%, ranging from 6.0 to 46.3%, with the average victimization rate being 33.08%, ranging from 13.99 to 57.5% (7). A national survey of South Korea found that 34% of students were involved in cyberbullying as bullies, victims or a combination of both (8). A similar study in Peru found that cyber-perpetration was ~5.6%, while the percentage of cyberbully-victims was around 17% (9).

Cyberbullying has been widely recognized as a public health concern with harmful impacts to those being bullied (10). Extant research has proven connections between cyberbullying and mental health problems (11), anti-social behaviors (12), and suicidal behaviors (13) among children and adolescents. Although many studies have attempted to estimate the prevalence of cyberbullying, their conclusions are always inconsistent. This is often due to the lack of diversity in data collected, such as the geographical distribution of participants (14). Among them, the differences in cyberbullying measurement scales adopted are likely to contribute significantly. This inconsistency appears unavoidable as each instrument is designed for a different purpose and, thus, their underlying constructs to be measured are different. Realizing the importance of cyberbullying instruments, scholars have shifted their attention to the development of cyberbullying measurements, focusing on their validation and application. A review of cyberbullying measurement tools in 2013 concluded that their reliability and validity were insufficient and called for more efforts to be made in creating unified standards and specific definitions (15). A follow-up systematic review, published in 2020, analyzed the fundamental features of cyberbullying measurement scales across 64 studies and emphasized the necessity for choosing reliable measurement scales for specific purposes (16).

Compared to adult groups, the children and adolescents may not well understand cyberbullying and specific measurement items if the survey questionnaires fail to address them clear enough for the youth to comprehensive. In this sense, the cyberbullying measurement capturing the youth experience requires more cautions. Although many instruments have been created and are available to assist in measuring specific cyberbullying behaviors or cyberbullying in general, it is still unclear their design purpose, corresponding scenarios, and their effectiveness for children and adolescents. To fill this research gap, this study aims to provide a comprehensive review of academic efforts on youth cyberbullying definitions and measurements, and their effectiveness. The research questions are as follows:

RQ1: In previous research, when scholars have examined different youth cyberbullying behaviors, did they provide a definition of cyberbullying behaviors and, if so, what was their definition?
RQ2: With advancements in social digital technologies, how have youth cyberbullying behaviors been classified and how has this affected their measurement?
RQ3: Have the previously proposed youth cyberbullying measurement scales worked effectively, and what was their popularity?

## Methods

This study followed the guidelines provided by Preferred Reporting Items for Systematic Reviews and Meta-Analyses (PRISMA) to conduct a systematic review that addresses the proposed research questions. Further, we traced the citations of important papers and employed a novel screening tool (i.e., ASReview), supported by active learning technology, to identify the most appropriated literature on youth cyberbullying.

### Data retrieval and screening

For data retrieval, this study consulted three widely used bibliographic citation databases, namely: Web of Science Core Collection, PubMed, and EBSCO. Since the first study identified pertinent to cyberbullying appeared in 2001, the date range of papers collected was limited to 1 January 2001 to 31 August 2021. The search terms used were divided into two parts, including cyberbullying and its measurement. The first part was retrieved using [cyberbullying OR cyber-bullying OR cyber-aggression OR ((cyber OR online OR electronic OR internet) AND (bully^*^ OR victim^*^ OR prepetrat^*^ OR violence OR threat^*^ OR harass^*^ OR aggression^*^ OR intimidate OR insult^*^ OR humiliate OR condemn^*^ OR isolate OR embarrass^*^ OR forgery OR slander^*^ OR flame OR stalk^*^ OR manhunt^*^))]. The second part was retrieved by (measurement^*^ OR measur^*^ OR scale^*^ OR questionnaire^*^ OR survey^*^ OR instrument^*^ OR (estimation method^*^)). We combined the two search strategies to obtain references on cyberbullying measurement. Our search focused on the papers' topic, title, and abstract, while the document type was limited to journals and conference papers. Initially, 1,654 papers were retrieved. After checking for any duplications, a total of 892 papers were retrieved for subsequent review.

To screen the retrieved papers, we employed an AI-assisted tool, ASReview, which is designed to improve screening efficiency for systematic reviews (17). ASReview requires users to specify all prior relevant and irrelevant papers to train the screening algorithm. Specifically, we randomly selected 10% of the documents for labeling at first, and then ASReview generated a reference list with a calculated relevance score to speed up the screening process.

**Figure 1:**
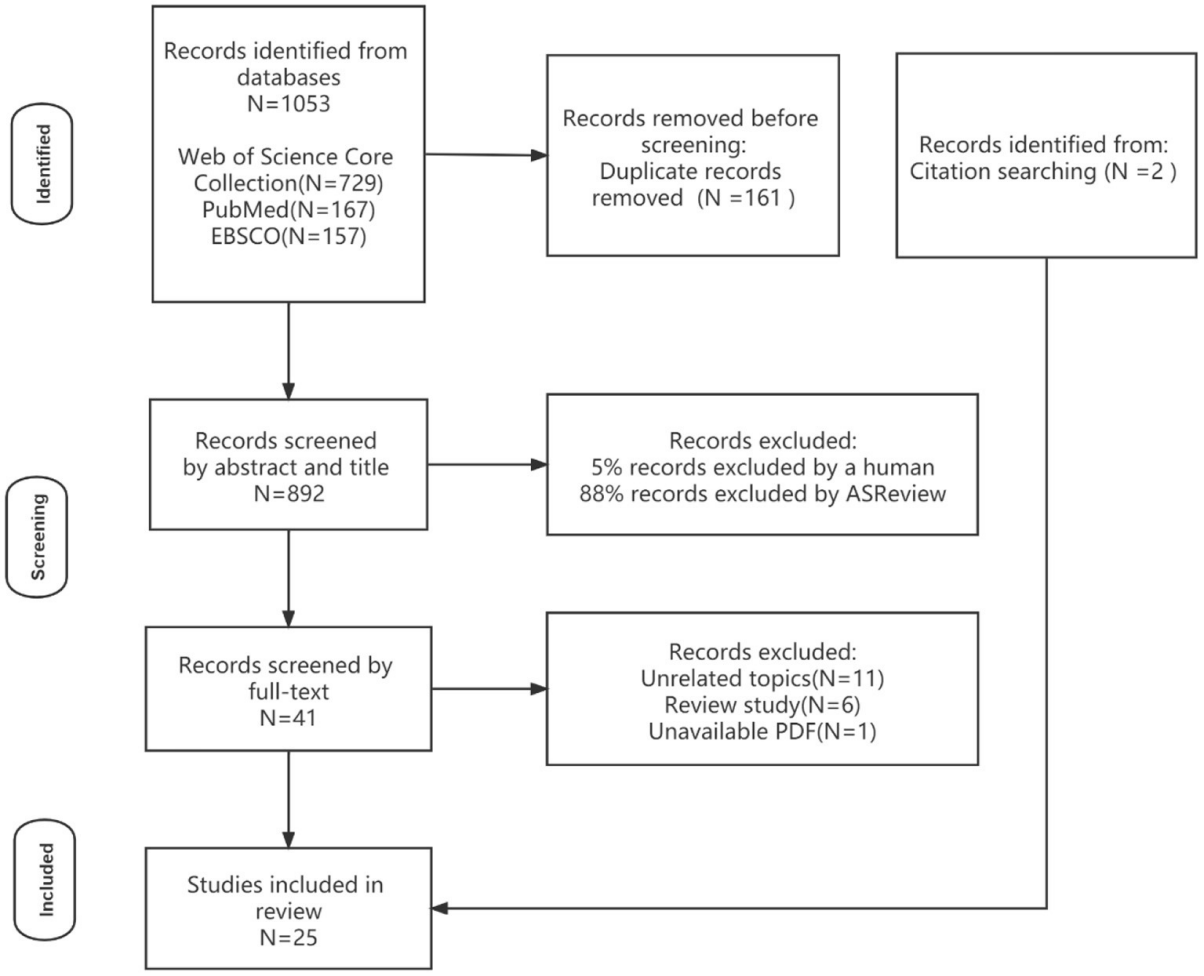
PRISMA flow chart for screening.

The study's selection criteria was as follows: (a) study targeted elementary school students, middle school students or college students; (b) the major theme of the paper was cyberbullying measurement; (c) the measurement scale proposed must highlight the cyberbullying scenario with representative terms, such as cyberbullying victimization, cyberbullying perpetration, online victimization, online aggression, e-victimization and e-bullying; (d) the paper must employ questionnaire survey as the main research method. The exclusion criteria was as follows: (a) studies targeting adults; (b) studies centered on workplace cyberbullying; (c) studies written in non-English language. After screening the titles and abstracts of all retrieved papers, a total of 40 remained. Subsequently, a more detailed full-text review of the 40 selected studies was completed. 11 studies were excluded for not concentrating on cyberbullying. 6 reviews were removed. 1 study was excluded due to unavailability of the full-text. In addition, we added two more studies from the cited references of retrieved papers. Finally, in total, we obtained 25 studies for review. Figure 1 presents the details of the final selected papers.

### Coding process

After the screening of papers, we extracted all necessary information to support subsequent analysis. Firstly, if the study specified the cyberbullying behaviors intended to be measured, we recorded the definition and highlighted the main construct. Secondly, we coded the cyberbullying types mentioned in the study and extracted the number of items, the factor structure of the measurement scales, and the content of the items. Finally, we coded the characteristics of the cyberbullying scales, focusing on its name, developers, users, year, sample size, respondents' age, period, psychometrics, and frequency of citation. For further details, please see Table 1 which shows the main types of cyberbullying and the characteristics of the cyberbullying scales.

**Table 1:** Youth cyberbullying scales.

**N**	**Cyberbullying instrument**	**Developer or user, year**	**Setting**	**Instrument classification (number of items)**	**Sample size**	**Age**	**Time frame**	**Psychometrics**	**Citations**
1	European Cyberbullying Intervention Project Questionnaire	Del Rey et al. 2015	Six European countries	CB (11 items); CV (11 items)	5,679	11 to 23 years old	N/A	CFI > 0.95, ECVI ≤0.6, McDonald's Omega = 0.99, Standardized Cronbach's alpha = 0.96	151
		Twardowska-Staszek et al. 2018	Poland	Same as above	1,052	9 to 16 years old	N/A	Satorra-Bentler chi-square = 981.92; df = 208; NFI = 0.98, NNFI = 0.98; CFI = 0.98; RMSEA = 0.06; Factor loadings(0.72, 0.91)	7
		Martinez et al. 2020	Peru	Same as above	607	12 to 19 years old	N/A	S/B chi-square = 583.28, df = 208, CFI = 0.95, NFI, 0.95, NNFI = 0.96, RMSEA = 0.058 (90% CI = 0.052–0.064)	6
		Williford et al. 2019	USA, online survey	CB (6 items); CV (6 items)		Grades 3 to 5	N/A	CFI = 0.596, alpha = 0.640	2
2	The Cyberbullying Triangulation Questionnaire	Gonzalez-Cabrera et al. 2019	Spain	CV (10 items); BY (10 items); CB (15 items)	5,036	10 to 23 years old	N/A	Omega >0.94; The measurement model is constant for the two age groups (10-14 years old and 15-23 years old)	13
		Gonzalez-Cabrera et al. 2020	Spain	CV (7 items) CB (7 items) BY (7 items)	2,068	11 to 19 years old	between 7 and 8 months	S–Bχ2 (165,N = 2,068) = 1,356.07; *p* < 0.001; RMSEA = 0.060, 95% confidence interval (0.054–0.065); CFI = 0.984; NNFI = 0.980; SRMR = 0.057; Factor loadings (0.61, 0.87)	6
3	The Florence CyberBullying-CyberVictimization Scales	Ersilia Menesini et al. 2011	Italy	CB (10 items); CV (10 items)	1,092	11-18 years old	In the past 2 months	Threshold 2 Monofactorial model CFI>0.95 RMSEA < 0.06	122
		Palladino et al. 2015	Italy	Written or verbal (7 items) visual (4 items) character impersonation (4 items) exclusion (3 items)	1,142	13 to 20 years old	In the last couple of months	CFI>0.917; RMSEA <0.026	30
4	The Cyberbullying and Online Aggression Survey Scale	Hamburger et al. 2011	USA	CV (1-6,9-12 items) CB (14-18 items)	-	N/A	In the last 30 days	N/A	N/A
		Brochado et al. 2017	Portugal		2,624	Grade 7 to 12	Lifetime, last 12 months, and the last 30 days	Cronbach's alpha: Victimization scale = 0.74 Offending scale = 0.76	2
5	The cyber Victimization Experiences and Cyber Bullying Behaviors scales	Betts et al. 2017	UK	CV (15 items) 1 Threats (6 items); 2 Sharing images (5 items); 3 Personal attack (4 items); CB (12 items) 1 Sharing images (4 items); 2 Gossip (5 items); 3 Personal attack (3 items)	393	11 to 15 years old	In the last three months	Cronbach's alpha of three factors of the victim scale were 0.91,0.88,0.85, respectively; Cronbach's alpha of CB were 0.86, 0.79, 0.81.	6
6	The Cybervictimization Questionnaire	David 2017	Spain	Character Impersonation (3 items); Visual or Sexual (3 items); Written or Verbal (6 items); Online exclusion (3 items)	3,159	12 to 18 years old	In the last three months	χ^2^/df = 3.14; CFI = 0.965; RMSEA [CI 90%] = 0.026 (0.023-0.029); Raykov's rho coefficient = (0.74-0.89)	15
7	Cyberbullying scale	Patchin et al. 2015	USA	CV (9 items); CB (9 items)	-	N/A	In the last 30 days	CV: Cronbach's alpha range 0.892–0.935; CB: Cronbach's alpha range 0.935–0.969;	107
8	The Online Victimization Scale	Tynes et al. 2010	USA	General online victimization (8 items); Sexual online victimization (6 items); Individual online racial discrimination (4 items); Vicarious online racial discrimination (3 items	N/A	14 to 19 years old	In the last year	Study 1: Theoretical four-factor model χ^2^(183) = 556.709; RMSEA = 0.096; TLI = 0.916; CFI = 0.927; IFI = 0.927; Study 2: χ^2^(183) = 570.303; RMSEA = 0.080; CFI = 0.939; IFI = 0.939	18
9	The Bullying and cyberbullying Scale for Adolescents	Thomas 2019	Australia	CV (5 items); CB (5 items)	1,217	12 to 17 years old	In the last 3 months	CV: CFI = 0.94; RMSEA = 0.07; CB: CFI = 0.94; RMSEA = 0.05	13
	The Bullying and cyberbullying Scale for Adolescents	Ozbey et al. 2020	Turkey	CV (5 items); CB (5 items)	600	12 to 18 years old	In the last 3 months	CVR ≥0.73; CV: KMO (0.500, 0.827); factor loads ≥0.630; Cronbach's Alpha internal consistency (0.606, 0.806) CB: KMO(0.500, 0.789) factor loads ≥0.679; Cronbach's Alpha internal consistency (0.616, 0.815)	0
10	Cyberbullying perpetration (CBP)? cyberbullying victimization (CBV)	Lee et al. 2017	USA	CB (20 items): 1Verbal or written (9 items); 2Visual or sexual (5 items); 3Social exclusion (6 items) CV(27 items): 1Verbal/written(10 items); 2Visual/sexual(10 items); 3Social exclusion(7 items)	286	18 to 25 year old	N/A	CBP Cronbach's alpha = 0.93; χ^2^/df = 1.97; CFI = 0.95; TLI = 0.94; RMSEA = 0.08; SRMR = 0.06 CBV Cronbach's alpha = 0.95; χ^2^/df = 2.86; CFI = 0.97; TLI = 0.95; RMSEA = 0.08; SRMR = 0.07	10
11	The Cyberbullying Questionnaire	Calvete 2010	Spain	16 items	1,431	12 to 17 years old	N/A	χ^2^(104, *n* = 1431) = 140; RMSEA = 0.016(0.0079, 0.022); NNFI = 1; CFI = 1 All factor loading ranges are between 0.90 and 0.99; Alpha coefficient is 0.96; The average correlation between items is 0.64	335
	N/A	Gamez-Guadix et al. 2014	Mexico	CB (14 items); CV (9 items)	1,491	12 to 18 years old	N/A	χ^2^(220, *N* = 1,491) = 293; *p* < 0.001; NNFI = 0.98; CFI = 0.99; RMSEA = 0.030 (95% CI: 0.027, 0.034); Factor loadings>0.59;	57
12	The Cyberbullying Scale	Stewart et al. 2014	USA	14 items	736	11 to 18 years old	In the last few months	EFA Factor Loading (0.72, 0.90); CFA Factor Loading (0.72, 0.90); Cronbach's alpha = 0.94	34
13	A self-report scale investigating	Pozzoli 2020	Italy	CB (4 items); CV (4 items); CD (4 items); BY (4 items)	561	11 to 15 years old	From the beginning of the school year	χ^2^(98) = 298.71; *p* < 0.001; CFI = 0.952; TLI = 0.941; RMSEA = 0.060 (90% CI: 0.053–0.068)	7
14	The E-Victimization Scale (E-VS) and the E-Bullying Scale (E-BS) for adolescents	Lam et al. 2013	China	CV (5 items); CB (6 items)	484	11 to 16 years old	In the last 7 days	E-VS single factor model: χ^2^/df = 13.580; RMSR = 0.034; GFI = 0.918; AGFI = 0.752; Factor loading (0.635, 0.854) E-BS two-factor model: χ^2^/df = 3.523; RMSR = 0.008; GFI = 0.963; AGFI = 0.902; Factor loading (0.315, 0.998)	28
15	An Internet-based survey	Patchin and Hinduja 2006	Online survey	Types of Online Bullying (7 items)	384	<18 years of age		N/A	1,354
16	A cyberbullying study	Smith et al. 2008	UK	Cyberbullying on seven media types	625	11 to 16 years old	In the last couple of months	N/A	1,463
17	Bullying and cyberbullying questionnaire	Monks et al. 2012	UK	CB (15 items) CV (15 items)	220	7 to 11 years old	N/A	N/A	74

Two authors were involved in the coding process. The first conducted the initial coding while the second checked the coding accuracy. Initially, the second author coded the 25 identified studies twice during two different periods and calculated the consistency. The kappa value was 0.84, indicating a high consistency. After that, the other authors randomly selected 5 studies out of the 25 to evaluate and identify possible deficiencies. Finally, the authors modified the coding scheme, made corresponding revisions, and agreed upon the final coding scheme.

## Results

### Descriptive analysis

**Figure 2:**
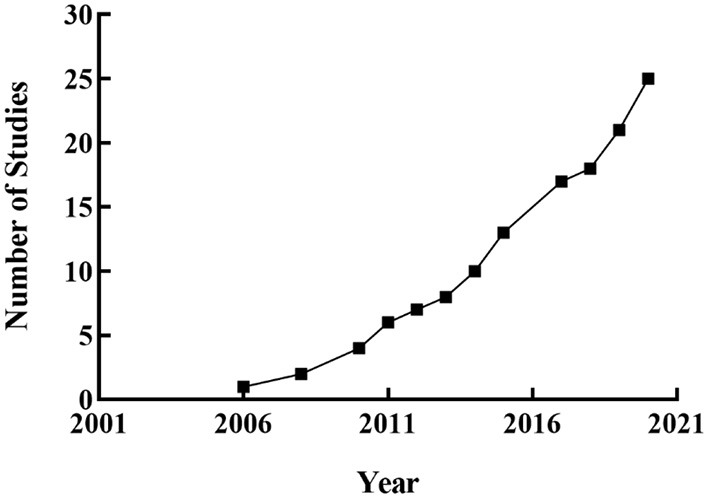
The trend in the number of reviewed studies from 2001 to 2021.

From the 25 reviewed papers, 24 were published as journal articles and 1 was published as a report. With regards time of publication, the earliest paper that met the study's selection criteria was published in 2006, with the number of studies increasing gradually since this date; Figure 2 illustrates the trend in publications by year. All papers employed a questionnaire survey approach. With regards the location of samples reported in the papers, 18 were from high income countries, including the United States (*N* = 6), Spain (4), Italy (3), the United Kingdom (*N* = 3), Australia (*N* = 1), Poland (*N* = 1), Portugal (*N* = 1), and Turkey (*N* = 1); the remaining papers reported on samples collected from 6 European countries (*N* = 1) and 3 other countries, including China (*N* = 1), Mexico (*N* = 1) and Peru (*N* = 1). 23 of the studies administered the questionnaire survey offline with only one employing an online questionnaire tool. From the 25 reviewed studies, 17 measurement scales were adopted. See Table 2 for details.

**Table 2:** The geographical distribution of respondents' locations in the reviewed studies.

**Location**	**Numbers of studies**
**Countries**	
USA	6
Spain	4
Italy	3
UK	3
Australia	1
China	1
Mexico	1
Peru	1
Poland	1
Portugal	1
Turkey	1
Six European countries	1
**Others**	
Online survey	1
Total	25

### Defining cyberbullying

In total, 11 studies provided respondents with a specific definition of cyberbullying prior to completion of the questionnaire; of these, seven different definitions were proposed. The definitions provided were similar as most studies loosely defined cyberbullying as using electronic communication to attack, make fun of, and harm others. In addition, two studies closely linked traditional bullying behaviors with cyberbullying behaviors when providing a definition. By using the classic definition of bullying, provided by Olweus and Limber (18), which emphasizes the three key elements of bullying (i.e., intention to harm, repetitiveness, and power imbalance), we further examined the definitions provided. In doing so, we found that, although some did not include the word “bullying” in their description, all highlighted the element of intention to harm. Meanwhile, four definitions mentioned the element of repetitiveness, and two referred to the element of imbalance of power; please see Table 3 for further details.

**Table 3:** Definitions provided in the reviewed studies.

**Definitions**	**Represent elements**	**Used in**
Cyberbullying is a new form of bullying, which involves the use of text messages, photos and videos, phone calls, e-mails, to attack another student (19).	IH	(20)
Cyberbullying is when someone repeatedly makes fun of another person online or repeatedly picks on another person through email or text message or when someone posts something online about another person that they don't like.	R, IH	(20)
Cyberbullying is when someone repeatedly harasses, mistreats, or makes fun of another person online or while using cell phones or other electronic devices.	R, IH	(21)
Disparaging remarks, symbols, images or behaviors that inflict harm through the use of computers, cell phones and other electronic devices (22).	IH	(23)
CBP is “directed toward an individual or a group using any form of electronic communications technology, such as the internet or mobile phones. “CBV is” being the object of aggressive or harmful behavior by others using any form of electronic communications device.”	IH	(24)
Cyberbullying is “an aggressive, intentional act carried out by a group or individual, using electronic forms of contact, repeatedly and over time against a victim who cannot easily defend him or herself” (3).	IH, IP, R	(25, 26)
A student is being bullied when another student or group of students does one or more of the following: • Says mean or hurtful things, makes fun of them, calls them names or threatens them. • Leaves them out of a group or an activity, or won't let them join in, on purpose. • Hits, kicks or pushes them around. • Spreads lies or rumors to make others not like them. • Uses the Internet or mobile phones to: * Send them mean or hurtful messages using words, pictures, or videos. * Send other people mean or hurtful messages about them. * Spread rumors/lies to make others not like them. * Leave a person out or not let them join in, on purpose. It is bullying when these actions happen again and again, and it is difficult for the person to defend themselves or make it stop happening. ** It is NOT bullying when teasing is done in a friendly and playful way. ** It is NOT bullying if two people who are as strong as each other argue or fight (27).	IH, IP, R	(27, 28)

IH, intention to harm; IP, imbalance of power; R, repetition.

From the 25 reviewed papers, only two included all three elements of bullying. The first, suggested by Smith et al. (3), defined cyberbullying as *an aggressive, intentional act carried out by a group or individual, using electronic forms of contact, repeatedly and over time against a victim who cannot easily defend him or herself*. This definition has been widely used in cyberbullying research due to its comprehensiveness and ease of understanding. The second, provided by Thomas et al. (29) was proposed when creating the Bullying and Cyberbullying Scale, and their definitions specify cyberbullying behaviors and emphasizes what is not cyberbullying (e.g., cyberbullying is NOT teasing, if done in a friendly or playful way). However, the definitions provided in most studies are insufficient, with several being too vague and lacking consistency. For example, the electronic methods of communication referred to are not always the same. Some refer to specific methods or mediums, such as text messages, and photos and videos, while others refer to any form of electronic communication. Another potential challenge is the characterization of the core elements of cyberbullying. Although some researchers suggest that cyberbullying is a subcategory of bullying, it is often difficult to reflect the three key elements of bullying in cyberspace.

### Types of cyberbullying

From the reviewed studies, two distinct types of cyberbullying emerged. The first focuses on the role of individuals while the second focuses on the aggressive behaviors demonstrated by individuals. In terms of individual roles, cyberbullying is frequently divided into two types, victims and perpetrators, while some later studies suggest a third type, namely cyberbullying bystanders. With regards the aggressive behaviors demonstrated by individuals, scholars most commonly identify four types, including written or verbal cyberbullying, visual cyberbullying, character impersonation, and exclusion. Similarly, others classify behaviors into more specific types, such as flaming (or roasting), harassment, denigration, defamation, outing, trickery, sexual harassment, and cyberstalking. For further details about how scholars classify the different roles and behaviors, please see Table 4.

**Table 4:** The foundations for cyberbullying classification.

**Classification basis**	**Category**	**Contents**
Individual role	Two/three categories	Cyberbullying victimization, cyberbullying perpetration, and bystanding
Nature of the offensive behavior	Four categories	Written or verbal, visual or sexual, character impersonation, and exclusion
Characteristics of the offensive behavior	Multi-category	Flaming (or roasting), harassment, denigration, defamation, outing, jokes, online sexual harassment, and cyberstalking

Cyberbullying victimization and perpetration are the most common classifications of cyberbullying suggested in related studies. In the early efforts of cyberbullying research, scholars investigated cyberbullying prevalence from the perspectives of both perpetration and victimization. Meanwhile, numerous studies have evaluated the experiences of victims, creating several instruments that are targeted toward cyberbullying victims (14). For example, Stewart et al. (25) developed the Cyberbullying Scale to comprehensively capture victims' experiences.

However, the suggested two-dimensional classification of cyberbullying fails to reveal the other factors that contribute to cyberbullying behaviors since it concentrates on victimization and perpetration only. Another string of research has explored the overlapping of the two types; for example, the bully-victim (11). Later, Gonzalez-Cabrera (30) added the overlooked role of bystanders and extended the current classification to three dimensions, including cyberbullying victimization, perpetration, and bystanders. If we take into account the overlapping of these three roles, seven roles are ultimately identified, including cyber victim, cyber bullying, bystanders, bully-victim, victims-bystander, bullying-bystander, and bully-victim-bystander. Subsequent research has also added the role of cyber defense, highlighting the behavior of internet users defending cyberbullying victims (31).

Among the reviewed studies, many classified cyberbullying as specific behaviors. For example, researchers defined and accepted four categories of cyberbullying behavior, including written or verbal behavior, visual behavior, rejection behavior, and character imitation behavior (19, 32). These behaviors are demonstrated in the Florence cyberbullying-cybervictimization scales (19, 29) and the results presented by Aizenkot and Kashy-Rosenbaum (33). In their efforts, the four types are specified as follows. The written or verbal type refers to individuals' behavior of using telephones, short messages, or e-mail to send offensive or insulting information to victims. Visual cyberbullying behavior refers to bullying through the publication of compromising or embarrassing pictures or videos by bullies. The exclusion type highlights the behavior of deliberately isolating individuals from online groups, such as online gaming groups or chat groups. The character imitation type refers to perpetrators pretending to be someone else by using victims' identities to communicate with others. This type of cyberbullying can be observed more frequently on social media platforms as users find it straightforward to create and use fake profiles. The imitation type may also cause considerable harm to teenagers and lead to unpredictable consequences. Attacks based on identity theft (i.e., using others' accounts to disclose personal information) is likely to bring significant pressure to sensitive teenagers as they are in a critical period of establishing their social identity. However, not all scholars recognize imitation as a cyberbullying behavior (24).

According to the characteristics of aggressive behaviors, cyberbullying can be further classified into the following types, including flaming (or roasting), harassment, denigration, defamation, outing, trickery, sexual harassment, and cyberstalking. The act of flaming (or roasting, as it is commonly known in the West) relates to posts that contains offensive, hostile, intimidating, insulting, satirical or unfriendly content. Typical behaviors include posting provocative or abusive posts to social media platforms with information often being characterized by extensive use of punctuation marks and capital letters (34). Harassment refers to the behavior of frequently sending offensive words to victims, such as repeatedly sending e-mails and deliberately disturbing the normal life of individuals. Denigration occurs when individuals' post and share content and, subsequently, perpetrators destroy the individual's reputation and interpersonal relationships by distributing distorting information, such as maliciously editing and uploading photos. Defamation is usually associated with denigration, but the former emphasizes the dissemination of false information *via* electronic communication. Dissemination of private information refers to the public sharing of others' secrets and embarrassing information by electronic communication (35). Although playing tricks often also involves the sharing of others' information, without their consent, it is mainly initiated by close contacts, such as friends or family members. With regards cyber sexual harassment, two types are frequently referenced. The first identifies the role of cyber sexual victimization which does not specify anyone in the sexual communication. The second is targeted toward individuals and encompasses the insulation of others' behaviors or gender, and requests for unwanted personal pictures, which are detrimental toward the personal and social development of children and adolescents. In addition, cyber sexual harassment can also be connected to illegal commercial activities, such as the deliberate publication of pornographic material without the consent of individuals (36). Cyberstalking relates to the tracking of victims' online activities with the intent of causing fear to the victim. This behavior is often repetitive and persistent (37). However, some studies prefer to call this type of behavior cyber harassment, cyber sexual violence, or cyberstalking, as they believe these affect adult groups only, instead of cyberbullying, which they believe is used only to describe affected adolescents.

### Cyberbullying instruments

The earliest instrument developed for measuring cyberbullying was proposed by Patchin and Hinduja (38). This preliminary study was conducted online and required participants to recall their cyberbullying experiences. The authors focused on seven cyberbullying behaviors, including the acts of ignoring, disrespecting, calling people names, threatening, picking on people, making fun of others, and spreading rumors. In realizing the differences in cyberbullying across media types, scholars have designed questionnaires to explore the variations by media type. For example, Smith et al. (3) designed an anonymous questionnaire, targeted at UK students aged between 11 to 16 years old, with the aim of evaluating the cyberbullying experiences of participants across seven electronic mediums, including telephone calls, text messages, emails, pictures or video clips, instant messaging, websites, and chatrooms. In a similar study, Calvete et al. (39) measured a wide range of cyberbullying behaviors through questionnaire, developing one of the earliest tools used to assess the cyberbullying prevalence among adolescents in Spain. The proposed instrument captured both cyberbullying victimization and cyberbullying perpetration, and satisfied existing psychometric standards. Building on their efforts, Tynes et al. proposed the Online Victimization Scale to specify the extent of online victimization in a multi-dimensional and comprehensive way. The authors employed confirmatory factor analysis to determine the factor structure of the online victimization scale and identified four different subscales, namely: general victimization, sexual harassment, personal racial discrimination, and alternative racial discrimination. The proposed scale was validated in Mexico and Spain respectively, and demonstrated that the scale had good model fit (23, 40).

From 2011 to 2016, seven representative scales were reported. The earliest was the Florence CyberBullying-CyberVictimization Scale which is a two-dimensional perspective that distinguishes cyber-perpetration and cyber-victimization as two independent modes. Menesini et al. (41) adopted Item Response Theory to analyze the severity and discrimination parameter of each behavior. However, the proposed scale has certain limitations, such as ignoring certain behaviors and media outlets, including isolation, character imitation, and cyberbullying on social media platforms. In later research, Palladino et al. (19) extended previous work by adding further items. However, the proposed scale was limited to Italy and required further comparison and verification in other countries to demonstrate its generalizability. Meanwhile, the Cyberbullying and Online Aggression Survey Scale, produced by the Centers for Disease Control and Prevention in the United States, has been widely adopted in many countries (20, 42). Apart from the scales designed for developed countries, Lam and Li (43) proposed the first scale which concentrated on developing countries, called the E-Victimization Scale (E-VS) and the E-Bullying Scale (E-BS). Both the E-VS and the E-BS have good model fit and discriminating capabilities and demonstrate positive significance on the evaluation of electronic bullying and victimization among Chinese youth, as well as possible comparisons for international cyberbullying prevalence and intervention. In addition, Stewart et al. (25) created the Cyberbullying Scale with their results showing strong psychometric characteristics. The authors suggested that the scale should be widely adopted to evaluate cyberbullying prevalence. Other important studies include the work completed by Monks et al. (26) who modified the bullying and cyberbullying questionnaire. Their contribution mainly the overlaps cyberbullying with traditional bullying.

Considering the absence of cross-cultural robustness analysis in collaborative research across countries, Del Rey et al. (44) proposed the European Cyberbullying Intervention Project Questionnaire (ECIPQ). The authors collected data from six European countries and found structural verification and cross-cultural robustness of the scale. Initially, the first version of the questionnaire was produced in English, but was subsequently translated into five languages and reverse-checked with the original version. The ECIPQ created a novel approach to undertaking cross-cultural surveys on cyberbullying. Upon the development of the scale, many scholars have duplicated and verified it in other countries. A study from the Peruvian Amazon affirmed that cyberbullying had similar characteristics and factor structures in both disadvantaged areas and wealthy countries (9). Another study from the United States used the ECIPQ to verify cyberbullying and victimization measures among adolescents at different ages (45).

From 2016, the number of cyberbullying scales developed has increased steadily. Considering the emergence of social digital technologies alongside the identified additional cyberbullying behaviors, Betts and Spenser (46) developed the cyber victimization experiences and cyberbullying behaviors scales. Meanwhile, they considered the existence of social desirability and the differences in adolescents' attitudes toward cyberbullying and posited that the specific items included in the CV and CB scales are not the same for capturing the subjective experiences of young people. Álvarez-García et al. further developed the Cybervictimization Questionnaire, highlighting four types of online victimization and four additional indicators (47). Their study affirmed the hypotheses of Nocentini et al. (32) regarding the multi-factor nature of cyber victimization, including written language, vision, rejection, and character imitation. In addition, the CyberBullying Perpetration (CBP) and CyberBullying Victimization (CBV) scales, proposed by Lee et al. provided an effective and reliable measurement structure for college students' cyberbullying experiences. It is an effective tool for evaluating the cyberbullying behaviors of young people during their early adulthoods. They validated three types of CBP and CBV, including verbal or written, visual or sexual, and social exclusion; their scale demonstrates strong psychometric characteristics. Building on this work, Gonzalez-Cabrera (30) proposed the Cyberbullying Triangulation Questionnaire which symbolized a breakthrough compared to traditional two-dimensional models of CB and CV. By adding the dimension of by-standing, it enabled researchers to evaluate the different perspectives of cyberbullying and examine the associations and possible overlaps between all cyberbullying roles. Pozzoli and Gini (31) offered a further extension of the role of bystanders' in cyberbullying. They verified the four-dimensional model of cyberbullying perpetration, victimization, defense, and passive bystander behavior, for the first time using a self-reporting questionnaire. Their results showed that the bullying behaviors in cyberspace are easily interchangeable. Considering this finding, some advocated that cyberbullying and bullying should be measured together. The authors developed a multidimensional measurement model based on behavioral forms, named the Bullying and cyberbullying Scale for Adolescents, which was used to examine the dimensional structure of the two related structures of traditional bullying and cyberbullying, separately (27).

### Scale evaluation

#### Target population

The samples analyzed in the reviewed studies predominantly included children, adolescents and early adults, ranging in age from 7 to 25 years old. In 15 of the 25 studies, respondents' ages ranged from 11 to 19 years old. The sample size ranged from several hundred to several thousand. The smallest sample size was 220 and the largest sample size was 5,679. 11 studies had a sample size of more than 1,000, while 6 studies had a sample size of <600.

#### Validity and reliability

Twenty-two studies reported statistical results of validity or reliability with sufficient psychometric support. Although the reported statistical indictors were not the same in all studies, internal consistency analysis, confirmatory factor analysis, and exploratory factor analysis methods, were used to test the reliability and validity of the proposed scales. All studies included had good psychometric characteristics. Firstly, among the 22 studies, 14 performed confirmatory factor analysis, where the CFI value of 13 studies was between 0.927 and 1.00, denoting good fit. However, the CFI value reported in one study was 0.596, denoting a poor fit; this study modified the ECIPQ and verified it using a sample of 7–11-year old respondents. The authors concluded that the ECIPQ scale was not sufficiently sensitive among young children (45). Secondly, 13 studies measured RMSEA. Among them, 12 studies had RMSEA values <0.08 while 1 study conducted two surveys where the RMSEA values were 0.087 and 0.096, respectively. Nevertheless, all the proposed models were considered acceptable. Thirdly, 9 studies examined the reliability of internal consistency. The Cronbach's alpha values of these were between 0.64 and 0.96. In addition, one study measured convergence validity and found that scale scores were positively correlated with indicators of anxiety, depression, and loneliness, and had significant statistical significance (25).

#### Scale adaptability

To examine the adaptability of existing cyberbullying scales, efforts have been made to replicate pilot studies in different regions and countries, and with those that speak different languages. The scale adaptability for cyberbullying has been extensively investigated through further verification and modification in recent years. For example, the European Cyberbullying Intervention Project Questionnaire has been applied to transnational and cross-cultural research in other regions, including Peru and Poland (9). Similarly, when using the Florence CyberBullying-CyberVictimization Scales, Palladino (19) took into account two further important cyberbullying behaviors, exclusion and imitation. The Cyberbullying and Online Aggression Survey Scale, proposed by the CDC, has also been verified in Portugal (42). This study shows that the proposed tools, reported on in the reviewed studies, have good psychometric properties, and that cyberbullying has similar characteristics and factor structures in different situations.

#### Scale popularity

The number of citations of the proposed measurement scales can reflect their acceptance by peers. We referred to the citation frequency of papers reported by Web of Science to demonstrate their popularity. The data was captured on the 27 October, 2021, and revealed that a sharp difference in citation frequency existed between the 17 proposed scales, at the first time of publication. Among them, the most frequently cited scales were those proposed prior to 2010. The scale with the highest number of citations was proposed by Smith et al. (3) with 1463 citations, followed by Patchin and Hinduja (38) with 1,354, and Calvete et al. (39) with 335. These three early explorations laid the foundations for the development of cyberbullying scales.

For newly developed scales, published after 2010, the European Cyberbullying Intervention Project Questionnaire, created by Del Rey et al. (44), the cyberbullying scale, produced by Patchin and Hinduja (38), and the Florence CyberBullying-CyberVictimization Scales, published by Menesini et al. (41) are all widely used, attracting more than 100 citations. These three scales are milestones in recent efforts. For instance, the ECIPQ has demonstrated a high adaptability across countries and regions. Based on the initial definition of cyberbullying, the newly developed cyberbullying scale, proposed by Patchin and Hinduja (38), analyzed the measurement and made a comprehensive evaluation, while the scale reported by Menesini (41), was the first to outline the structure of cyberbullying and its different types, in the context of Italian youth cyberbullying and cyber victimization.

## Discussion

Scholars have actively engaged in the development of cyberbullying measurement scales during the last 20 years. This signifies the prevalence of cyberbullying across the world and makes comparisons among different regions and countries possible (48). Although cyberbullying *via* social media platforms has become a more salient topic, the knowledge gap is still visible among the public, which calls for more measurement with adaptability across countries. To address this concern, many efforts have been made to create measurement scales that focus on youth cyberbullying in the last 20 years (47). This study reviewed a total of 25 studies, including 17 representative cyberbullying scales, and explored the characteristics and development trends of the measurement scales.

### The variations of cyberbullying definition

Alongside the evolution of cyberbullying behaviors trigged by the emergence of social digital technologies, scholars have begun to design novel scales to capture new cyberbullying behaviors derived from their original definition (16, 49). Although the key elements of cyberbullying have been widely acknowledged, the behaviors targeted by scholars' may vary by study. There is no wonder, therefore, that cyberbullying scales have developed quickly in recent years. Due to the variations in focus, cyberbullying scales appear not always consistent, in terms of the measurement structure. As a consequence, the reliability of cyberbullying prevalence rates, indicated in research, as well as their policy implications, has remained questionable (29). For example, our review indicates that scholars have not always followed the classic definition of cyberbullying during measurement. In addition, advancements in social digital technologies are reshaping how scholars' conceptualize cyberbullying which requires caution when drawing comparisons. For example, the high ownership of smartphones globally makes it difficult to compare differences between mobile phone based cyberbullying and internet cyberbullying (19). With regards survey methods, this review highlights the challenge of providing a clear and concise definition for young respondents. Ideally, respondents should be able to easily understand the definition provided and share the same understanding with survey researchers. However, this is not always the case. For example, when providing a definition for cyberbullying, some prefer to avoid the term *bullying*, using alternatives such as *harassment, intimidation and tormenting* or omitting the term entirely. This is likely to lead to variations in the estimation of cyberbullying prevalence. Meanwhile, some scholars have offered a comprehensive list of specific cyberbullying behaviors as a complement to the provided definition. Similarly, without a proper definition of the term *bullying*, it can lead to a higher prevalence estimation and capture more experiences. One study that compared the results of different measurements found that measurements without definitions, including the term *bullying*, reported a higher rate of cyberbullying victimization, compared to those that included the term *bullying* in the introduction (50).

By acknowledging the overlap between bullying and cyberbullying, demonstrated in empirical studies, some authors proposed a more precise measurement that captured traditional bullying and cyberbullying in parallel (27, 51). Based on a cross-national survey, 45.8% of respondents that reported cybervictimization also reported traditional victimization (51). Measuring the act of cyberbullying, therefore, requires additional standards, such as online publicity and anonymity (29). Publicity and anonymity *via* the internet may play more important roles than repetition and power imbalance which characterizes cyberbullying. In addition, future studies should examine the potential impact of publicity and anonymity on the power imbalance of cyberbullying.

### Refining efforts in cyberbullying measurement

This review shows a trend in the refinement of cyberbullying behaviors and their corresponding measurements. The efforts made have gradually increased the number of cyberbullying classifications, from one type to various types and have increased the number of measurement items, as a result. There are a couple of reasons for this. Firstly, compared with a single item instrument, the psychometrics of an instrument with multiple items are higher in terms of reliability and validity. Meanwhile, multiple items capture richer behaviors and make the exploration of cyberbullying factor structure possible (52). Secondly, studies have increasingly adopted behavioral questionnaire design as an approach to reducing the influence of social desirability and cultural differences. Questionnaires have avoided stigmatization by asking questions related to specific aggressive behaviors without referring to children and adolescents as bullies or victims. Considering the differences in defining cyberbullying across social and cultural backgrounds, the instruments concentrating on specific cyberbullying behaviors are helpful for cross-national studies. In addition, the definitions provided in many studies are either too conceptual or too operational with the ambiguity making the effectiveness of measurement questionable (16). Due to the complexity and severity of cyberbullying, many scholars have adopted a multidimensional approach with more detailed explanations being provided to aid consistency and provide more reliable measurements (29). This wave in refinement of measurements has been accelerated by the claims that bullying and cyberbullying should be examined together while more contextual theory and experiences of adolescent aggression need to be taken into account (52). With growth in online hate activities, Tynes et al. (23) developed the Online Victimization Scale focused on racial discrimination online. This makes cyberbullying measurement even more complicated.

In addition, the advancement and reliability of social digital technologies may contribute to this trend. With regards reliability, questionnaires with good psychometric features in a single country or region need to be adjusted and verified in other countries or regions to affirm their reliability (53). As understanding of cyberbullying may vary across countries with variations in culture and language, translated questionnaires may have semantic differences in wording and cultural differences in the concept (54).As for advancements in social digital technologies, scholars must constantly update their conceptualization and measurement instruments. Many instruments have been verified in both developed and developing countries, and by using different age groups among the youth as samples; for example the Florence CyberBullying-CyberVictimization Scales, European Cyberbullying Intervention Project Questionnaire, the Cyberbullying and Online Aggression Survey Scale, and the Bullying and Cyberbullying Scale for Adolescents (9, 19, 27, 28, 41, 42, 44).

### Promoting public awareness of youth cyberbullying

In realizing the prevalence of cyberbullying among the youth, the topic has gradually attracted attention from scholars across multiple disciplines, including psychology, sociology, epidemiology, communication, computer science, and management science. Although scholars have proposed numerous cyberbullying scales that involve multiple types to determine possible cyberbullying behaviors, not all netizens demonstrate sufficient awareness. Some teenagers tend to describe cyberbullying as “just words” and believe it does not affect their happiness (55). Poor understanding of cyberbullying can easily affect the reliability and accuracy of the measurement. Despite the fact that law and regulations have been created and put into practice in the United States, the United Kingdom, and other countries, many teenagers may still not know when they have violated the law (56). In fact, only sensational cases on youth cyberbullying attract youth attention, while the rest become buried on the internet (57).

In this vein, unraveling the undesired consequences of cyberbullying helps to increase youth awareness of cyberbullying. Existing measurement scales seldom examine the consequences of cyberbullying in terms of intensity and the extent of the harm caused to victims. Few scholars regard consequences as a basis to identify cyberbullying, as well as the severity of cyberbullying. Although a recent review found that the prevalence of cyberbullying has increased in recent years, it remains unknown their intensity and associated harm (7, 58). In addition, variations in intensity and their associated impact has resulted in different types of cyberbullying which require further investigation.

### Limitation and future implications

Our review critically examines efforts in youth cyberbullying measurement, including cyberbullying definitions, cyberbullying types, and the formation of specific measurement scales. Our results call for a consistent definition of cyberbullying, which is critical in the structured measurement of youth cyberbullying. The 17 scales reported are developed for different reasons and try to capture specific cyberbullying behaviors or the experiences of young respondents. This review can help scholars in youth cyberbullying choose appropriate measurement scales accordingly.

Nevertheless, this review is not flawless. Although the screening process was assisted by a novel machine learning tool, ASReview, we cannot guarantee the inclusion of all appropriate measurement scales. Potential biases should also be noted. For instance, the papers from Asian countries have low presentations, which may either attribute to the language searched or the adoption of the ASReview. Future research can duplicate our efforts with manual review and compare with our results. However, our review does demonstrate that efficiency of ASReview adoption in the screening process for systematic reviews. It is suggested, therefore, that future systematic reviews also employ this tool to further explore its effectiveness. In addition, we focused on the questionnaire survey exclusively. However, the youth cyberbullying is cyber-based behaviors, and alternative measurement methods may also worth future attention. For instance, future studies can capture youth behaviors by collecting social media data directly, and thus estimate cyberbullying frequency, specify subsequent consequences as well as corresponding coping strategies (59, 60).

## Conclusion

This study provides a comprehensive review of academic efforts on cyberbullying definitions and measurements, and their effectiveness, during the last 20 years. Specifically, we examine existing cyberbullying measurements for children and young adolescents. In total, 25 studies, mentioning 17 cyberbullying measurement scales, met the study collection criteria and were subsequently reviewed. Our findings revealed that most studies failed to provide a clear definition of cyberbullying, often providing unclear and inconsistent descriptions to respondents of survey questionnaires. In general, the definitions provided found it difficult to incorporate the three key elements of bullying into their description, including the act of being harmful, repetitive, and the power imbalance between bullies and victims. With regards the classification of cyberbullying, most reviewed studies suggested two or three categories, including cyberbullying victimization, cyberbullying perpetration, and bystanding. Some also proposed four types, based on the nature of the aggressive behavior, including written or verbal, visual or sexual, character impersonation, and exclusion. In addition, cyberbullying behaviors included flaming (or roasting), harassment, denigration, defamation, outing, jokes, online sexual harassment, and cyberstalking. As for measurements, many scales have been proposed during the last 20 years and have been subsequently refined. Many have been revised or improved more than once and have been verified in different countries and using different ages of samples.

## Data availability statement

The raw data supporting the conclusions of this article will be made available by the authors, without undue reservation.

## Author contributions

WZ, SH, and CZ conceived the study. WZ and SH contributed to the data collection and wrote the draft. WZ, SH, LL, CZ, and RE contributed to the data analysis. WZ, CZ, LL, and RE contributed to the discussion and revision. All authors have approved the submission.

## Funding

This research was supported by National Natural Science Foundation of China (72104087).

## Conflict of interest

The authors declare that the research was conducted in the absence of any commercial or financial relationships that could be construed as a potential conflict of interest.

## Publisher's note

All claims expressed in this article are solely those of the authors and do not necessarily represent those of their affiliated organizations, or those of the publisher, the editors and the reviewers. Any product that may be evaluated in this article, or claim that may be made by its manufacturer, is not guaranteed or endorsed by the publisher.
