# Supplementary material for: Cyberbullying definitions and measurements in children and adolescents: Summarizing 20 years of global efforts
Source: Front Public Health. 2022 Oct 25;10:1000504. doi: 10.3389/fpubh.2022.1000504 (PMC9642089; doi:10.3389/fpubh.2022.1000504)
Supplement: Supplementary file 1 [file Data_Sheet_1.PDF]

Appendix 1 Characteristics of the 25 included studies

N	Title	Author, Year	Cyberbullying Instrument	Setting	Sample size	Age
1	Structural validation and cross-cultural robustness of the European Cyberbullying Intervention Project Questionnaire	Del Rey et al. 2015	European Cyberbullying Intervention Project Questionnaire	Six European countries	5679	11 to 23 years old
2	Bullying and cyberbullying in Polish elementary and middle schools: Validation of questionnaires and nature of the phenomena	Twardowska-Staszek et al. 2018	European Cyberbullying Intervention Project Questionnaire	Poland	1052	9 to 16 years old
3	Bullying and Cyberbullying in Adolescents from Disadvantaged Areas: Validation of Questionnaires; Prevalence Rates; and Relationship to Self-Esteem, Empathy and Social Skills	Martinez et al. 2020	European Cyberbullying Intervention Project Questionnaire	Peru	607	12 to 19 years old
4	Validation of a Cyber Bullying and Victimization Measure Among Elementary School-Aged Children	Williford et al. 2019	European Cyberbullying Intervention Project Questionnaire	USA, online survey	841	Grades 3 to 5
5	Psychometric properties of the cyberbullying triangulation questionnaire: A prevalence analysis through seven roles	Gonzalez-Cabrera et al. 2019	The Cyberbullying Triangulation Questionnaire	Spain	5036	10 to 23 years old
6	Psychometric properties of the triangulated version of the European Bullying Intervention Project Questionnaire: Prevalence across seven roles	Gonzalez-Cabrera et al. 2020	The Cyberbullying Triangulation Questionnaire	Spain	2068	11 to 19 years old
7	The measurement of cyberbullying: dimensional structure and relative item severity and discrimination	Ersilia Menesini et al. 2011	The Florence CyberBullying-CyberVictimization Scales	Italy	1092	11-18 years old
8	Psychometric Properties of the Florence CyberBullying-CyberVictimization Scales	Palladino et al. 2015	The Florence CyberBullying-	Italy	1142	13 to 20 years old

N	Title	Author, Year	Cyberbullying Instrument	Setting	Sample size	Age
			CyberVictimization Scales			
9	Measuring bullying victimization, perpetration, and bystander experiences: a compendium of assessment tools	Hamburger et al. 2011	The Cyberbullying and Online Aggression Survey Scale	USA	-	
10	Cyberbullying Among Adolescents: The Influence of Different Modes of Inquiry	Brochado et al. 2017	The Cyberbullying and Online Aggression Survey Scale	Portugal	2624	Grade 7 to 12
11	Developing the Cyber Victimization Experiences and Cyberbullying Behaviors Scales	Betts et al. 2017	The cyber Victimisation Experiences and Cyber Bullying Behaviours scales	UK	393	11 to 15 years old
12	Validation of the Cybervictimization Questionnaire (CYVIC) for adolescents	David, 2017	The Cybervictimization Questionnaire	Spain	3159	12 to 18 years old
13	Measuring cyberbullying: Implications for research	Patchin et al. 2015	Cyberbullying scale	USA	-	
14	The Development and Validation of the Online Victimization Scale for Adolescents	Tynes et al. 2010	The Online Victimization Scale	USA		14 to 19 years old
15	Development and validation of the Bullying and Cyberbullying Scale for Adolescents: A multi‐dimensional measurement model	Thomas, 2019	The Bullying and cyberbullying Scale for Adolescents	Australia	1217	12 to 17 years old
16	Psychometric properties of the Turkish version of the Bullying and Cyber Bullying Scale for Adolescents (BCS-A)	Ozbey et al. 2020	The Bullying and cyberbullying Scale for Adolescents	Turkey	600	12 to 18 years old
17	Validation of Measures of Cyberbullying Perpetration and Victimization in Emerging Adulthood	Lee et al. 2017	Cyberbullying perpetration (CBP) and	USA	286	18 to 25 year old

N	Title	Author, Year	Cyberbullying Instrument	Setting	Sample size	Age
			cyberbullying victimization (CBV)			
18	Cyberbullying in adolescents: Modalities and aggressors' profile	Calvete, 2010	The Cyberbullying Questionnaire	Spain	1431	12 to 17 years old
19	Psychometric Properties of the Cyberbullying Questionnaire (CBQ) Among Mexican Adolescents	Gamez-Guadix et al. 2014	The Cyberbullying Questionnaire	Mexico	1491	12 to 18 years old
20	The Development and Psychometric Investigation of the Cyberbullying Scale	Stewart et al. 2014	The Cyberbullying Scale	USA	736	11 to 18 years old
21	Behavior during cyberbullying episodes: Initial validation of a new self‐report scale	Pozzoli, 2020	A self-report scale investigating	Italy	561	11 to 15 years old
22	The validation of the E-Victimisation Scale (E-VS) and the E-Bullying Scale (E-BS) for adolescents	Lam et al. 2013	The E-Victimisation Scale (E-VS) and the E-Bullying Scale (E-BS) for adolescents	China	484	11 to 16 years old
23	Bullies Move Beyond the Schoolyard A Preliminary Look at Cyberbullying	Patchin and Hinduja, 2006	An Internet-based survey	Online survey	384	Less than 18 years of age
24	Cyberbullying: Its nature and impact in secondary school pupils	Smith et al. 2008	A cyberbullying study	UK	625	11 to 16 years old
25	The emergence of cyberbullying: A survey of primary school pupils' perceptions and experiences	Monks et al. 2012	Bullying and cyberbullying questionnaire	UK	220	7 to 11 years old
